# Nicotine Impairs the Response of Lung Epithelial Cells to IL-22

**DOI:** 10.1155/2020/6705428

**Published:** 2020-02-29

**Authors:** Hannah My-Hanh Nguyen, Jaclene Amber Torres, Sudhanshu Agrawal, Anshu Agrawal

**Affiliations:** Division of Basic and Clinical Immunology, Department of Medicine, University of California, Irvine, Irvine, CA 92697, USA

## Abstract

Smoking is a major risk factor for pulmonary diseases that include chronic obstructive pulmonary diseases (COPD) and cancer. Nicotine is the toxic and addictive component of tobacco products, like cigarettes, that negatively affects the immune system. Here, we examined the effect of nicotine on the IL-22 pathway that protects lung function by increasing transepithelial resistance and epithelial cell regeneration and repair. Our results indicate that exposure to nicotine impairs the regenerative capacity of primary bronchial epithelial cells in scratch assays. IL-22 at 100 ng/ml significantly improved wound healing in epithelial cells; however, the exposure to nicotine hampered the IL-22-mediated effect of wound healing. Investigation into the mechanisms showed that IL-22 receptor, IL-22R*α*1, was downregulated in the presence of nicotine as determined by q-PCR and flow cytometry. We also investigated the effect of nicotine on IL-22 production by T cells. Results indicate that nicotine inhibited the secretion of IL-22 from T cells in response to aryl hydrocarbon receptor (AHR) ligand, FICZ. Altogether, the data suggests that nicotine negatively influences the IL-22-IL-22R axis. This impairment may contribute to the nicotine-mediated detrimental effects on lung function.

## 1. Introduction

Smoking has immediate and long-term consequences that negatively affect the respiratory and immune system. It is a major risk factor for pulmonary diseases including chronic obstructive pulmonary diseases (COPD), idiopathic pulmonary fibrosis (IPF), acute respiratory distress syndrome (ARDS), asthma, and cancer [1–5]. Nicotine is the toxic chemical that makes tobacco products addictive. Nicotine is also the primary culprit affecting lung health and immunity. It enhances the inflammatory response of macrophages and neutrophils but suppresses the phagocytic capacity as well as the release of reactive oxygen species by both these cell types [6, 7]. The proliferation of lymphocytes as well as cytotoxic activity is both inhibited in the presence of nicotine [8]. Furthermore, nicotine also has a profound effect on epithelial cell functions. Respiratory epithelial cells play an important role in lung defense. They form a barrier preventing entry of harmful and pathogenic substances. In addition, epithelial cells secrete mucus and antimicrobial peptides for host defense. The maintenance of epithelial cell health is an integrative part of pulmonary well-being [9–11]. Chronic nicotine exposure has been reported to affect mucociliary transport as well as increase in goblet cell hyperplasia. Enhanced lung inflammation and suppression of apoptosis by nicotine is the major cause of damage to the respiratory epithelium as it enhances cell transformation and survival leading to cancer [10, 11]. Though the effect of nicotine on epithelial cells has been studied in detail by numerous investigators, the influence of some newly emerged mediators such as IL-22, which play a role in epithelial cell health, is not well understood.

IL-10 family member cytokine, IL-22 has emerged as a major player in pulmonary defense and repair [12–15]. IL-22 is produced primarily by immune cells such as CD4^+^ T cells in response to infections and aryl hydrocarbon receptor ligands [16]. However, the receptor for IL-22 is expressed on nonimmune cells such as the fibroblasts and epithelial cells of the lungs [17]. IL-22 has been demonstrated to display an important role in maintaining lung homeostasis as well as in fighting infections. When produced in conjunction with IL-17, it synergizes with IL-17 in host defense against extracellular bacteria and fungi via enhancing the secretion of antimicrobial peptides and other mediators. However, IL-22 alone plays an important role in homeostatic processes in the lung epithelial tissues. It enhances AEC proliferation, which helps in regeneration and repair both at homeostasis and injury after infection. IL-22 has been reported to elevate the transcription of genes, which can protect the mucosal tissue against damage and improve wound healing [12]. It acts as a mitogenic stimulus for epithelial cells. IL-22 increases transepithelial resistance by utilizing mechanisms that instruct leukocytes and induce the regeneration of bronchial epithelial tissues [18]. In addition, IL-22 prevents apoptosis by producing antiapoptotic proteins which was shown to be protective in the bleomycin model of lung injury [17]. In another report, IL-22 was found to increase autophagy in alveolar epithelial cells. Autophagy protects AECs from stress as autophagy-deficient mice displayed increased lung pathology in response to respiratory syncytial viral infection. Furthermore, the AECs from these mice produced increased levels of IL-1*β* [19]. IL-22 plays a key role in defense against *Klebsiella pneumoniae* and *Citrobacter rodentium* in and fungal infections in the lung and the intestine [12]. Decrease in IL-22 is associated with enhanced fibrosis is observed in the lung during *Bacillus subtilis* infection [20]. Furthermore, both IL-17 and IL-22 support the release of metalloproteinases (MMPs), which facilitate the migration of immune cells to the site of inflammation by inducing the proteolytic degeneration of collagens and proteoglycans [21]. Emerging evidence indicates a role of IL-22 in the pathology of COPD, IPF, ARDS, and cancer. For example, COPD patients have increased numbers of IL-22^+^ cells in the bronchial mucosa compared with controls [22]. Similarly, neutralization of IL-22 resulted in elevated numbers of CD4^+^ T cells and acceleration of lung fibrosis in IPF [23]. Furthermore, accumulating evidence also suggests that IL-22 plays a critical role in regulating collagen deposition in the lung [20]. IL-22 is also reported to promote proliferation and metastasis of lung cancer cells, and the levels of the cytokine are reported to be increased in non-small-cell lung cancer patients. Interestingly, IL-22 exerts a protective role in ARDS by promoting lung repair [23].

There is a key gap in knowledge regarding the effect of smoking induced changes in the secretion of IL-22 and its role in resolution and repair in the lung. In light of the critical role of IL-22 in regulating immunity and inflammation in the AECs, its impairment by nicotine may contribute to the respiratory problems and diseases prevalent in the smokers. The intent of this study was to investigate the effect of nicotine on IL-22 production and response.

## 2. Materials and Methods

### 2.1. Cell Culture and Nicotine Exposure

Normal primary human bronchial epithelial cells, referred to as airway epithelial cells (AECs), were obtained from Lonza (Walkersville, MD). The AECs were from nonsmoking 28-year-old male donor and 24-year-old female donor. The cells were cultured in media supplied by the manufacturer as described [24, 25]. A549, lung epithelial cell line was obtained from American Type Culture Collection (ATCC, Manassas, VA) and cultured in RPMI 10% FBS containing glutamine, penicillin, and streptomycin. Cells between passages 5-8 were used for the experiments. Nicotine (N-3876) in liquid form was obtained from Millipore-Sigma (St. Louis, MO).

### 2.2. Epithelial Cell Repair Assay

The scratch assay was used to measure cell migration during *in vitro* wound healing [26]. Cells were grown to 95% confluence on a 12-well culture plate. Subsequently, they were stimulated with or without 1-10 *μ*M of nicotine and varying concentrations of IL-22 (0-100 ng/ml). Cells with nicotine were stimulated for 72 hours, and nicotine-containing media were replenished every day. Afterward, a 1 ml pipette tip was used to create a scratch, and then the cells were stimulated with IL-22. To measure the progression of wound healing, culture dishes were observed every day and micrographs were taken at 72 hours using a microscope with the AmScope program. Pictures were always taken from the same area in all dishes.

To analyze the scratch assay's micrographs, the area of the scratch was measured using the ImageJ and MRI wound healing tool. The MRI wound healing tool setting was set to find edges with a radius measured at one, unless the measurements required manual bordering. To measure the percentage of wound healed, the micrograph taken after 72 hours from the scratch was compared with the day the scratch was made. The students were blinded during the taking as well as analysis of the photographs.

The cells were stimulated with nicotine for 72 hours and underwent its respective experimental protocols. Scratch test with A549 and AECs was performed a minimum of three times with each cell line.

### 2.3. q-PCR for IL-22R*α*1

The cells were stimulated with nicotine (1-10 *μ*M) for 72 hours. Total RNA was extracted from cells using TRIzol reagent (Invitrogen) as per the manufacturer's protocol. The cDNA was prepared, and q-PCR was performed using the gene-specific primer for human IL-22R*α*1 (forward, 5′-CCCCACTGGGACACTTTCTA-3′ and reverse, 5′-TGGCCCTTTAGGTACTGTGG-3′) and GAPDH (forward, 5′-GTCTCCTCTGACTTCAACAGCG-3′ and reverse, 5′-ACCACCCTGTTGCTGTAGCCAA-3′) as internal control. Relative gene expression was quantified by normalizing Ct values with the corresponding GAPDH.

### 2.4. Flow Cytometry

The treated human AECs were collected by scraping since treatment with trypsin led to the disappearance of the IL-22R*α*1. Cells collected were washed with PBS 2% FBS staining buffer by centrifugation. The resulting pellet was suspended in PBS 2% FBS and surface stained with specific antibodies for IL-22R*α*1 PE (Clone #305405) and IL-28R*α* Alexa 647 (Clone #601106) (R&D Systems, Minneapolis, MN) for 30 min. Specific isotype controls were used both receptors. Cells were acquired using BD FACSCalibur and analyzed by the FlowJo software (Ashland, OR).

### 2.5. IL-22 Production by PBMCs

PBMCs from healthy controls were stimulated with endogenous aryl hydrocarbon receptor (AHR) ligand, 6-formylindolo [3,2-b] carbazole (FICZ) [27, 28] in the presence of anti-CD3 and CD28 beads (Dynabeads, Thermo Fisher, Carlsbad, CA). After six days, supernatants were collected for estimation of cytokines, IL-22, IL-17, and IFN-*γ* by specific ELISAs. The cells were stimulated with PMA and ionomycin and brefeldin A for 4 h. Subsequently, the cells were collected and washed and surface stained for CD4 using CD4 PerCP antibody (Clone #OKT4) (BioLegend, San Diego, CA). After washing, the cells were fixed and permeabilized using Cytofix/Cytoperm kit (BD Biosciences, San Juan, CA). Intracellular cytokine staining (ICC) for IL-22 was performed using IL-22 PE antibody (Clone #2G12A41) (BioLegend, San Diego, CA). Cells were acquired using BD FACSCalibur and analyzed by the Flow Jo software (Ashland, OR).

### 2.6. Statistical Analysis

Using GraphPad Prism software, the experimental data were analyzed with repeated measures one-way ANOVA and interpreted at a 95% confidence interval (*α* = 0.5).

## 3. Results

### 3.1. Nicotine Inhibits the Repair Capacity of Lung Epithelial Cells

**Figure 1:**
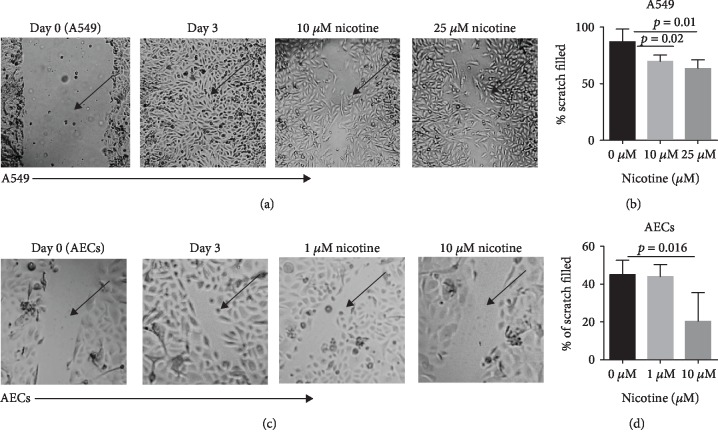
Nicotine inhibits the repair capacity of lung epithelial cells. A549 or AECs exposed to nicotine at various concentrations for 72 h were scratched, and the capacity of the cells to fill scratch was determined. Quantitation of percent scratch filled was calculated using the ImageJ software. (a) Scratch repair at 72 h in A549 cells. (b) Quantitation of the same. (c) Micrographs display the scratch repair at 72 h in AECs. (d) Quantitation of the same. Data is presented as the mean ± SD of biological replicates of *n* = 4 for A549 and *n* = 5 for AECs. Arrows point to the scratches. Scale for micrograph is 20 *μ*m.

The present study explores the effect of nicotine in lung injury. We assessed the repair capacity of lung alveolar epithelial cell line, A549, using the scratch assay model. The scratched cells were treated with different concentrations of nicotine ranging from 10-25 *μ*M. Nicotine was obtained in solution form (6.23 M) from Sigma and diluted to the required concentration in the culture mediums for both cells. The concentration used was based on the literature [10, 29, 30]. As shown in micrograph (Figure 1(a)), the presence of nicotine at both 10 *μ*M and 25 *μ*M prevented the healing of the scratch compared with controls. Quantitation of biological replicates of the image indicated that healing is significantly reduced on 10 *μ*M (*p* = 0.01) and 25 *μ*M (*p* = 0.023) nicotine treatment (Figure 1(b)). Next, we confirmed our observations using normal human primary bronchial epithelial cells (AECs). Here, we used lower concentrations of nicotine since initial experiments did not exhibit a difference between 10 and 25 *μ*M nicotine concentrations. Similar to A549, nicotine at 10 *μ*M significantly inhibits the wound healing (Figure 1(c)) in AECs. AECs display approximately 45% healing at 72 h which is significantly reduced to approximately 15% (*p* = 0.03) on treatment with nicotine (Figure 1(d)). Nicotine at 1 *μ*M was comparable with controls. Taken together, these results indicate nicotine inhibits the regenerative and repair capacity of both A549 and AECs.

### 3.2. Nicotine Inhibits IL-22-Mediated Epithelial Cell Repair

**Figure 2:**
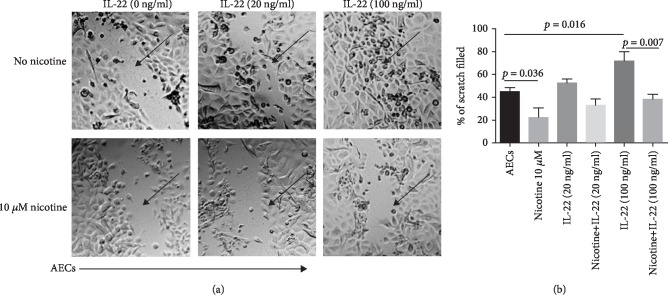
Nicotine inhibits IL-22-mediated epithelial cell repair. Primary AECs were grown to 95% confluence on 12-well culture dishes that were then scratched and treated with IL-22+ (with)/- (without) nicotine 10 *μ*M (Nic) at the concentrations shown. (a) Photomicrographs shown are 72 h later. (b) Quantitation of the data. Data is presented as the mean ± SD of 5 experiments using AECs from 2 different donors. Arrows point to the scratches. Scale for micrograph is 20 *μ*m.

Since IL-22 has been reported to help epithelial cell regeneration and repair [31, 32], we investigated whether addition of IL-22 can overcome the nicotine-mediated inhibition of repair in AECs. As expected, the presence of IL-22 accelerated the scratch repair at 72 h. The concentration of 100 ng/ml was more effective than 20 ng/ml of IL-22 in filling the area (Figure 2(a)). There is approximately thirty percent or more healing as compared with control (Figure 2(b)). In contrast, AECs exposed to nicotine displayed reduced recovery comparable with control AECs even in the presence of IL-22 at 100 ng/ml (Figures 2(a) and 2(b)). These data indicate that nicotine inhibits the capacity of AECs to respond to IL-22 and repair the wound.

### 3.3. Downregulation of IL-22R Expression on AECs by Nicotine

**Figure 3:**
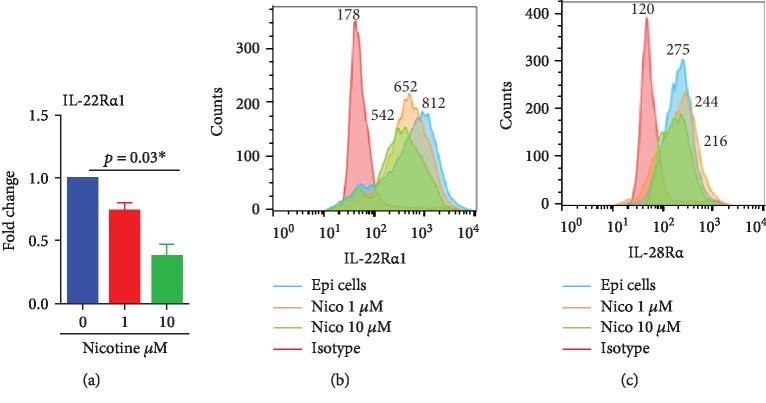
Downregulation of IL-22R expression on AECs by nicotine. Primary human AECs were exposed to nicotine 1-10 *μ*M for 72 h. The expression of IL-22R*α*1 as determined by (a) q-PCR. ∗ indicates that *p* value is significant. (b) Flow cytometry. (c) Expression of IL-28r*α* by flow cytometry. Data is representative of 4 experiments with AECs from 2 different donors. Numbers on the histograms depict the MFI.

To investigate the mechanisms underlying reduced response of nicotine exposed AECs to IL-22-mediated effects, we determined the effect of nicotine on IL-22 receptor. The IL-22 receptor is a type 2 cytokine receptor comprised of the heterodimeric complex with IL-22R*α*1 and IL-10R2 [33, 34]. While IL-10R2 is constitutively expressed in cells throughout the body, IL-22R*α*1 is expressed almost exclusively in epithelial tissues [35]. Interestingly, IL-22 has been shown to have no affinity for IL-10R2 and a very high affinity to bind to IL-22R*α*1 [36–38]. Binding of IL-22 to IL-22R*α*1 increases its affinity for IL-10R2 [35–38], suggesting a stepwise process in the binding [39]. Since IL-22R*α*1 is the primary receptor on AECs, q-PCR and flow cytometry were used to measure the expression of IL-22R*α*1. Nicotine exposure led to a concentration-dependent decrease in IL-22R*α*1 gene expression at 72 h with significant differences being observed at 10 *μ*M concentration (Figure 3(a)). Flow cytometry was used to confirm the gene expression data. As it is evident from Figure 3(b), AECs displayed abundant expression of IL-22R*α*1 that was downregulated in a concentration-dependent manner in nicotine-exposed cells with significant downregulation observed at 10 *μ*M nicotine. In addition to IL-22R*α*1, we also examined the expression of IL-28R*α* because IL-28 (IFN-*λ*1) also uses the IL-10R2 subunit along with IL-28R*α* for signaling [40]. Furthermore, the expression of IL28R*α* is also restricted to cells of the epithelium [41]. In contrast to IL-22R*α*1, the expression of IL-28R*α* was comparable between nicotine-treated and nicotine-untreated AECs (Figure 3(c)) indicating that suppression of IL-22R*α*1 is specific. These data indicate that nicotine may be inhibiting the action of IL-22 by suppressing the expression of its receptor on AECs.

### 3.4. AHR Ligand, FICZ, Induces IL-22 Production in PBMCs from Healthy Subjects That Is Suppressed by Nicotine

**Figure 4:**
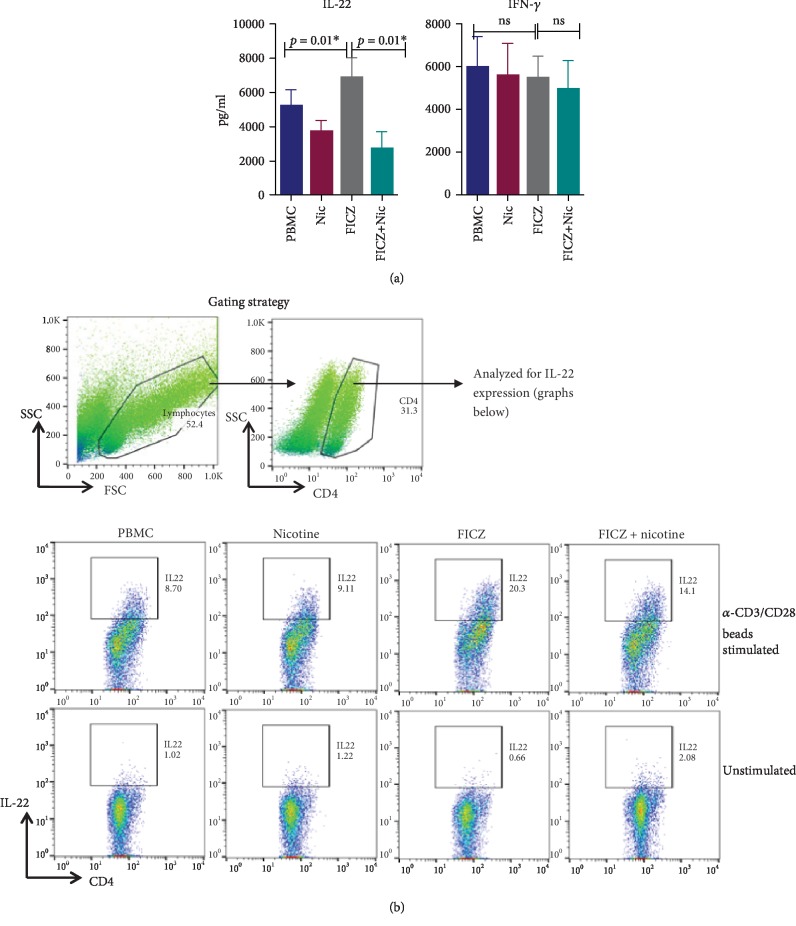
AHR ligand, FICZ, induces IL-22 production in PBMCs from healthy subjects which is suppressed by nicotine. Anti-CD3 and CD28 bead-stimulated PBMCs were exposed to FICZ, nicotine, or both for 5 days. (a) ELISA for IFN-*γ* and IL-22. Result is presented as the mean ± SD of 4 experiments. ∗ indicates that *p* value is significant. (b) Gated CD4 T cells were analyzed for IL-22 expression by intracellular cytokine staining (ICC) using flow cytometry. Green dot plots depict the gating strategy. PBMCs without bead stimulation are shown as controls. Data is representative of 4 experiments.

Given the importance of IL-22 in maintaining respiratory health, we examined the production of IL-22 as well as IL-17 and IFN-*γ* by T cells from healthy subjects in response to aryl hydrocarbon receptor (AHR) ligand, FICZ. FICZ is an endogenous ligand of AHR that has been demonstrated to induce IL-22 in human T cells [27, 28]. Since stimulation of PBMC with anti-CD3 and CD28 in the presence of FICZ is known to induce high levels of IL-22, we used PBMCs for our experiments. As expected, anti-CD3 and CD28 stimulation of PBMCs in the presence of FICZ resulted in significantly increased induction of IL-22 in the FICZ-treated PBMCs compared with controls. Addition of nicotine (10 *μ*m) suppressed IL-22 production from FICZ-treated PBMCs (Figure 4(a)). There was a reduction in IL-22 secretion by nicotine in untreated PBMCs, but the difference was not significant. FICZ did not induce IFN-*γ* at significant levels over PBMC controls, and suppression by nicotine was not significant (Figure 4(a)). IL-17 was below the detection limit (data not shown). Intracellular cytokine staining (ICC) was performed to confirm that CD4^+^ T cells are the source of IL-22 is being produced. The percentage of IL-22-producing cells was significantly lower in the nicotine-treated group that was stimulated with FICZ. No IL-22 expression was observed in PBMCs unstimulated with anti-CD3/CD28 beads. These data indicate that nicotine suppresses AHR ligand-induced IL-22 production in PBMCs, particularly in CD4 T cells.

## 4. Discussion

In the present study, we demonstrate that nicotine significantly affects the IL22 axis. It suppresses the response of AECs to IL-22 by downregulating the expression of the receptor for IL-22 and IL-22R*α*1. Furthermore, nicotine also inhibits the production of IL-22 by T cells in response to the AHR ligand, FICZ.

Chronic exposure to cigarette smoke (CS) is known to modulate the epithelial cell barrier functions as well as suppress immunity, resulting in increased susceptibility to respiratory infections [11, 42–44]. Recent reports have highlighted the pivotal role of IL-22/IL-22R signaling in epithelial cells at barrier surfaces in the initiation, regulation, and resolution of immune responses. Most of the studies are focused on examining the effect of cigarette smoke on production IL-22, and only a few reports have examined its effect on the receptor for IL-22 in epithelial cells. One study had investigated the expression of IL-22R*α*1 on the epithelial cells in lung tissue from both murine and human COPD subjects and controls. They observed approximately a twofold decrease in *IL-22R1* protein expression in the lungs of cigarette smoke-exposed mice compared with control animals, while the gene level was not affected [45]. Cleavage of IL-22R by proteases from neutrophils was considered the primary mechanism. We find a decrease in the expression of IL-22R*α*1 both at the gene and protein level in nicotine exposed AECs (Figures 2 and 3). Thus, it is possible that nicotine is suppressing the receptor via a different mechanism. Ubiquitination and degradation of IL-22R*α*1 have been reported in murine epithelial cells, and expression of GSK-3*β* is thought to play a role. Depletion of GSK-3*β* depletion in cells was shown to reduce the level of the IL-22 receptor [46]. Interestingly, nicotine and its major metabolite, cotinine, have also been reported to inhibit GSK3-*β* [47].

In addition to downregulating the IL-22R in AECs, nicotine also inhibits the production of IL-22 from CD4^+^ T cells in response to endogenous AHR ligand, FICZ. AHR is an evolutionary conserved receptor that acts as a sensor for chemical pollutants, xenobiotics, and other harmful inhaled substances [48]. Several endogenous ligands for AHR have also been identified. These are mainly metabolites of aromatic amino acids such as tryptophan, including FICZ [16]. FICZ has been reported to play a crucial role in normal physiological processes where it helps in maintaining the barrier function and providing resistance to bacterial infections [16]. CS has been demonstrated to activate the AHR and upregulate cytochrome P4501A1 (*Cyp1a1*) mRNA even with low concentrations. The presence of polycyclic aromatic hydrocarbons (PAHs) in the cigarette smoke is considered the major culprit [49, 50]. The effect of nicotine alone on AHR activation in PBMCs has not been examined. Our data indicates that nicotine by itself may suppress the activation of AHR as we observe a downregulation of IL-22 production with FICZ.

Nicotine signals primarily via nicotinic receptors (nAChR) and has been reported to modulate various immune cell functions including proliferation, differentiation, migration, and cell-cell interactions [51, 52]. CD4^+^ T cells display ubiquitous expression of the receptor, *α*7nAChR [53]. Activation of nAChR*α*7 by nicotine in CD4^+^ T cells was found to suppress Th17 responses while blocking the receptor with an *α*7nAChR inhibitor (*α*-BTX) partially restored the IL-17 expression [54].

Studies from the literature examining the effect of cigarette smoke in several disease models also support our findings. Pichavant et al. observed an impairment in the production of IL-22 and to some extent IL-17 in response to *S. pneumoniae* in a mouse model of COPD induced by chronic CS exposure [55]. The group also reported similar findings *ex vivo* in COPD patients [55]. In fact, IL-22 defect was considered as a key factor in COPD exacerbations, both in patients and in the murine model. Similarly, in another study also with chronic CS exposure COPD model, decreased production of IL-22 was associated with delayed clearance of NTHi bacteria and enhanced airway remodeling compared with controls. Addition of IL-22 was able to enhance bacterial clearance and limit lung damage in CS-exposed mice [56]. In contrast to infectious disease models, CS was found to enhance IL-22 and IL-17 responses in autoimmune diseases. In a mice model of rheumatoid arthritis (RA), exposure to cigarette smoke was demonstrated to upregulate the production of IL-22 by *Def6^−/−^* DO11.10 CD4^+^ T cells. They reported that cigarette smoking inhibited the activation of ROCK2 activation and prevented the phosphorylation of IRF4, a negative regulator of IL-22 production [57]. In the same study, no significant effect was observed on IL-17 and IL-21 production, the two other cytokines produced by Th17 cells. An increase in IL-17 and IL-22 was also found in CS-exposed T cells from psoriatic patients [58]. Thus, it seems when cells are already polarized towards Th17 as in the autoimmune diseases, CS enhances IL-17 and IL-22 secretion, but in infectious disease models where IL-22 needs to be induced, CS suppresses the production of IL-22.

In summary, the results of this study indicate that nicotine profoundly affects the IL-22-IL-22R axis which is a vital link between immunity and barrier function. To our knowledge, this is the first report that examines the effect of nicotine on this axis. This information can potentially be used for therapeutic or diagnostic purposes for pulmonary diseases related to smoking.

## Data Availability

Data will be made available on request.
